# Transcript Profiling of *Hevea brasiliensis* during Latex Flow

**DOI:** 10.3389/fpls.2017.01904

**Published:** 2017-11-07

**Authors:** Jinquan Chao, Shuguang Yang, Yueyi Chen, Wei-Min Tian

**Affiliations:** Ministry of Agriculture Key Laboratory of Biology and Genetic Resources of Rubber Tree and State Key Laboratory Breeding Base of Cultivation and Physiology for Tropical Crops, Rubber Research Institute, Chinese Academy of Tropical Agricultural Sciences, Danzhou, China

**Keywords:** *Hevea brasiliensis* Muell. Arg., duration of latex flow, latex metabolism, gene expression, qRT-PCR

## Abstract

Latex exploitation enhances latex regeneration in rubber trees. The latex exploitation-caused latex flow lasts from 10 min to a few hours, which is convenient for exploring the transcript profiling of latex metabolism-related genes at the different stages of latex flow. In the present study, the expression pattern of 62 latex metabolism-related genes involved in water transportation, carbohydrate metabolism, natural rubber biosynthesis, hormone signaling, ROS generation and scavenging, and latex coagulum across three stages of latex flow between rubber tree clones CATAS7-33-97 and CATAS8-79 were comparatively analyzed by quantitative real-time PCR. The two clones show differences in latex regeneration and have a different duration of latex flow. The results showed that the expression levels of 38 genes were significantly higher in CATAS8-79 latex than in CATAS7-33-97 during latex regeneration, while 45 genes had a notably higher expression level in CATAS8-79 latex during latex flow. Together with the activation of the MEP pathway and jasmonate pathway in CATAS8-79 latex, *HbPIP1;3, HbPIP1;4, HbSUT3, HbSus3, HbHMGS1-2, HbMK* should contribute to the high latex regeneration ability. The up-regulation of ethylene signaling and *Hb44KD* and the down-regulation of latex coagulation-related genes in CATAS8-79 latex might contribute to its longer latex flow duration. This study provides some cues for revealing the regulation of latex metabolism in rubber trees.

## Introduction

Natural rubber (*cis*-1,4-polyisoprene) is an essential industrial substance around the world. Due to its high yield and excellent physical properties, the para rubber tree (*Hevea brasiliensis*) is the main source of natural rubber (Eschbach and Lacrotte, 1989). Laticifers located at the inner bark of rubber tree serve as the location of natural rubber biosynthesis. Laticifer cells have a specialized cytoplasm containing 30–50% rubber for natural rubber refinement (Chrestin et al., 1997). In natural rubber production, latex is collected by severing the laticifer rings every 2–3 days. This process is termed tapping (d’ Auzac, 1989). After tapping, 10 to a few 100 ml of latex are expelled from the severed laticifers. The latex flow is terminated by plug formation at the end of the laticifer’s wounded site after several minutes to a few hours after tapping. The duration of latex flow after tapping is one of the crucial factors that determines the rubber yield of the rubber tree. It is influenced by multiple factors such as ethylene application, temperature, latex redox homeostasis, etc. (Zhu and Zhang, 2009; Chao et al., 2016). Ethylene is a phytohormone that regulates numerous developmental as well as physiological processes in plants (Van de Poel et al., 2015). Ethrel, an ethylene releaser, is widely used to increase the latex production per tapping since it can significantly prolong the duration of latex flow (Zhu and Zhang, 2009).

It is well known that latex exploitation enhances latex regeneration in the laticifer cells within the drainage areas. Latex regeneration is a complex molecular reconstruction process, which is not only involved in *de novo* protein synthesis, but also in the rebuilding of the lost organelles, such as rubber particles, lutoids and ribosomes, etc. The duration of the latex flow after tapping usually lasts several minutes to a few hours. Although latex regeneration occurs at two tapping intervals, it should be initiated by the latex flow. Available data show that several rubber biosynthesis-related genes and homologues of the transcriptional complex genes significantly fluctuated during latex flow (Chao et al., 2016). Exploring the transcript profiling of latex metabolism-related genes at different stages of latex flow will provide some new cues about the regulation of latex regeneration (the early stage of latex flow) and latex flow (the late stage of latex flow).

Both the latex production and the duration of latex flow are much higher in rubber tree clone CATAS8-79 than in rubber tree clone CATAS7-33-97. CATAS8-79 originated from the offspring of a CATAS88-13 and CATAS217 cross, while rubber tree clone CATAS7-33-97 arose from the offspring of a RRIM600 and PR107 cross. The changes in the overall latex production between the two clones may be associated with the difference in latex regeneration and the duration of latex flow after tapping. In the present study, 62 genes involved in water transport regulation, carbohydrate metabolism, rubber biosynthesis, jasmonate and ethylene signaling, ROS generation and scavenging and latex coagulation were analyzed by qRT-PCR between CATAS8-79 and CATAS7-33-97 during latex flow following tapping. The results provide an initial transcript profiling of latex metabolism, which is beneficial to understand the mechanism for latex regeneration and latex flow in rubber trees.

## Materials and Methods

### Plant Materials

Eleven-year-old rubber tree clones, CATAS7-33-97 and CATAS8-79, with the same circumference were used in the present study. The trees were grown at the Experimental Station of the Rubber Research Institute at the Chinese Academy of Tropical Agricultural Sciences in Danzhou city, Hainan province. These trees were regularly tapped for latex collection using a half spiral pattern, every 3 days, without Ethrel stimulation (S/2, d/3). Ten trees of each clone were selected and tapped in an S/2 d/3 system. After tapping, the latex samples were collected at 1, 30, and 60 min for CATAS7-33-97, and at 1, 80, and 150 min for CATAS8-79, which respectively represented the early, middle, and late stage of the latex flow. Each of the three batches of latex samples were individually collected from ten trees of each clone, and placed on ice for determination of the rubber content in the latex or stored at -80°C for total RNA extraction (Chao et al., 2015a).

### RNA Isolation and cDNA Synthesis

Total RNA was extracted using the protocol of RNAprep pure Plant Kit protocol (Tiangen, China). The concentration and quality of RNA were examined by NanoDrop 2000 (Thermo Scientific Inc., United States), and the integrity of the RNA samples was checked by 1.5% agarose gel electrophoresis. Synthesis of cDNA was performed using the RevertAid^TM^ First Strand cDNA Synthesis Kit (Fermentas, Canada) following the manufacturer’s protocol.

### qRT-PCR Analysis

The expression pattern of 62 latex metabolism-related genes (*Hb44KD, HbAACT1-3, HbACO1-2, HbAPX1, HbCAT, HbChit, HbCMK, HbCOI1, HbCuZnSOD, HbDXR, HbDXS1-2, HbEIN2-3, HbETR1-2, HbFDPS, HbGluc, HbHDR, HbHDS, HbHevein, HbHMGR1, HbHMGS1-2, HbHRT1-2, HbIPPI1, HbJAZ2-3, HbLOX, HbMCT1-2, HbMDC1-2, HbMDS1-2, HbMnSOD, HbMK, HbMYC1, HbMYC3, HbNIN1-3, HbPDC4, HbPIP1;3-4, HbPIP2;1,3,5,7, HbPK, HbPMK, HbREF, HbRBOHA-B, HbSAMS, HbSRPP, HbSus3 and HbSUT3*) reported in the previous study were used here (Tang et al., 2010; He, 2013; Xiao et al., 2014; An et al., 2015; Chao et al., 2015b; Liu et al., 2015; Long et al., 2015; Putranto et al., 2015; Deng et al., 2016; Shi et al., 2016; Makita et al., 2017). Reactions were carried out in 384-well plates as follows: 95°C for 3 min followed by 45 cycles of 95°C for 15 s, 60°C for 60 s and 72°C for 30 s, and a melting curve from 55 to 95°C, which increased by 0.5°C every 30 s. Each real-time PCR reaction was performed in triplicate. The Bio-Rad CFX384 Manager 3.0 software was used for visualizing and analyzing the data, including the quantification cycle values and the efficiency of PCR reactions. The relative expression levels of target genes were normalized with HbUBC2b (Chao et al., 2016), and displayed using a heat map. All primer pairs used in this article were list in Supplementary Table S1.

### Rubber Content Determination

For rubber content determination, 100 μl of acetic acid were dropped into 1 g of fresh latex to obtain the rubber coagula. The rubber coagula were washed in water for 2 h, then dried overnight at 55°C and weighed. The experiments were repeated three times (Chao et al., 2015a).

### Statistical Analysis

For multiple group comparisons, statistical analysis was performed with SPSS Statistics 17.05 using the analysis of variance (ANOVA) based on Duncan’s test. The capital letter represents *P* < 0.01, while the lower case letter represents *P* < 0.05. The same letter indicates no significant difference among groups. For two group comparisons, statistical analysis was performed with GraphPad Prism 5 based on *T*-test.

## Results

### Determination of Rubber Content in Latex during Latex Flow

**FIGURE 1:**
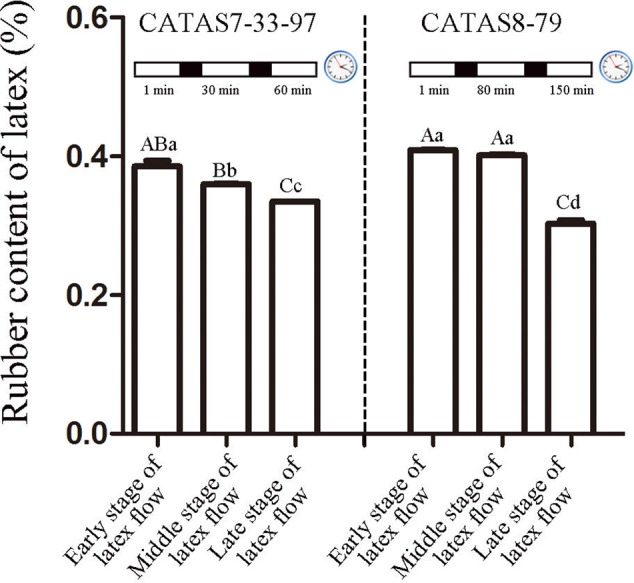
Rubber content of latex across three stages of latex flow between CATAS7-33-97 and CATAS8-79. The capital letter represents *p* < 0.01 while lower case represents *p* < 0.05. The same letter indicated no significant difference among groups.

The duration of latex flow in rubber tree clone CATAS7-33-97 was approximately 70 min, which was much shorter than that in rubber tree clone CATAS8-79 (more than 160 min) (Chao et al., 2016). Here, the rubber content in the latex during latex flow was determined. Changes in the rubber content of both clones occurred during the latex flow (**Figure 1**). A differential fluctuation pattern of the rubber content was apparent between the two clones (**Figure 1**). The decrease in the rubber content in the latex of CATAS7-33-97 was significant at the middle stage (30 min) (*P* < 0.05) and highly significant at the late stage (60 min) (*P* < 0.01) of latex flow. In contrast, there was no significant difference in the rubber content of the latex of CATAS8-79 between at the early stage (1 min) and the middle stage (80 min), but there was at the late stage (150 min) when the rubber content showed a highly significantly decreased (*P* < 0.01) and was significantly lower (*P* < 0.05) than the rubber content in the latex of CATAS7-33-97 at the late stage of latex flow (**Figure 1**).

### Expression Pattern of Water Transport-Related Genes during Latex Flow

**FIGURE 2:**
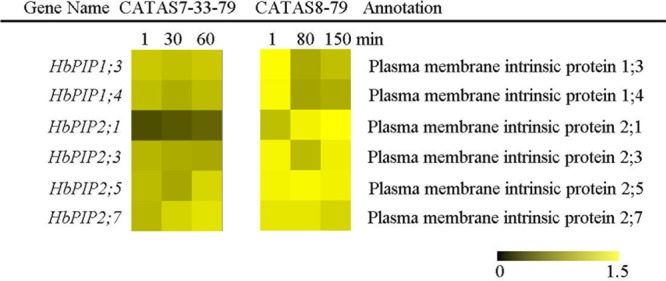
The qRT-PCR analysis of aquaporin encoding genes across three stages of latex flow between CATAS7-33-97 and CATAS8-79. The qRT-PCR result was displayed by heatmap. Yellow showed high expression and black showed low expression. Bar represented the relative expression data (the same below).

Upon tapping, aquaporin controls the water entering the laticifer cells and plays a crucial role in both latex flow and latex regeneration (Zou et al., 2015). The expression of six water transport-related genes (*HbPIP1;3, HbPIP1;4, HbPIP2;1, HbPIP2;3, HbPIP2;5* and *HbPIP2;7*) was analyzed during the latex flow (**Figure 2** and **Supplementary Figure S1**). Among the tested six genes, the expression of most genes had no obvious difference in the latex of CATAS7-33-97 except for *HbPIP2;5* and *HbPIP2;7* which were significantly up-regulated at the late stage of the latex flow. The differentially activated expression of most genes was present in the latex of CATAS8-79 during latex flow. Of these, the *HbPIP1;3, HbPIP1;4* and *HbPIP2;3* were significantly up-regulated at the late stage of latex flow and their expression levels were higher than that of the corresponding genes at the early stage of latex flow in CATAS7-33-97. Additionally, *HbPIP2;1* was up-regulated at the middle stage and at the late stage of latex flow in both clones.

### Expression Pattern of Carbohydrate Metabolism-Related Genes during Latex Flow

**FIGURE 3:**
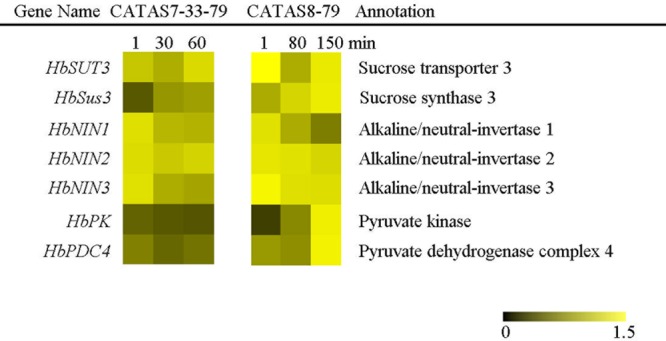
The qRT-PCR analysis of carbohydrate metabolism-related genes across three stages of latex flow between CATAS7-33-97 and CATAS8-79.

In plants, carbohydrate metabolism, such as sucrose formation and degradation through glycolysis, provides both energy and a carbon skeleton for organic compound formation (Kunz et al., 2014). The expression of all the tested seven sucrose metabolism and glycolysis related genes changed in the latex of both clones during latex flow (**Figure 3** and **Supplementary Figure S1**). In general, the expression pattern of *HbNIN1, HbNIN2* and *HbNIN3* was similar between the two clones. Their expression levels were high within 1 min (at the early stage of latex flow) and decreased thereafter during latex flow. The levels of *HbSUT3* at the early stage and *HbSus3* at the late stage in the latex of CATAS8-79 were respectively higher than those in the latex of CATAS7-33-97, although their expression patterns were similar between the two clones. In contrast to the expression pattern of *HbPK*, which was down-regulated in the latex of CATAS7-33-97 during latex flow, it was up-regulated in the latex of CATAS8-79 during latex flow. Similarly, the expression level of *HbPDC4* was significantly higher at the late stage in the latex of CATAS8-79 than in the latex of CATAS7-33-97.

### Expression Pattern of Natural Rubber Biosynthesis-Related Genes during Latex Flow

**FIGURE 4:**
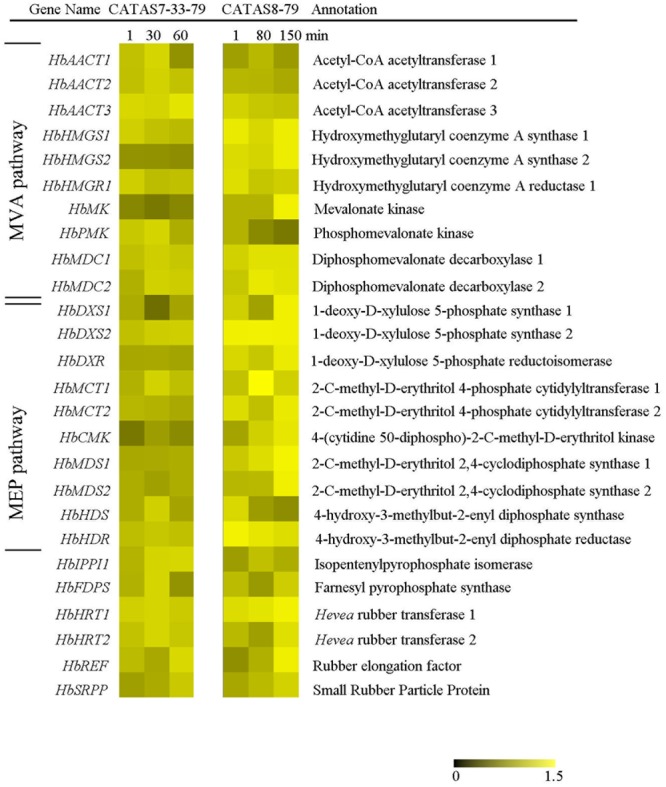
The qRT-PCR analysis of natural rubber biosynthesis metabolism-related genes across three stages of latex flow between CATAS7-33-97 and CATAS8-79.

Isopentenyl pyrophosphate is the direct precursor for natural rubber (Chow et al., 2012). The expression of 26 genes related to pre-IPP and post-IPP stages of the natural rubber biosynthesis pathway were analyzed during latex flow by qRT-PCR (**Figure 4** and **Supplementary Figure S1**). The expression of nearly all the tested genes changed during latex flow. Of these, the transcript abundance of 13 genes (*HbHMGS1, HbHMGS2, HbMK, HbMDC1, HbMDC2, HbDXS1, HbDXS2, HbDXR, HbMCT2, HbCMK, HbMDS1, HbHDR* and *HbSRPP*) at both the early and late stages in the latex of CATAS8-79 were higher than those in the latex of CATAS7-33-97. Moreover, the expression pattern of 15 genes, *HbAACT1, HbHMGS1, HbHMGS2, HbHMGR1, HbMK, HbMDC1, HbMDC2, HbDXS1, HbMCT1, HbMCT2, HbHDR, HbIPPI1, HbHRT1, HbREF* and *HbSRPP*, were generally similar between the rubber tree clone CATAS7-33-97 and CATAS8-79. The other seven genes had differential expression patterns during the latex flow between the two clones. They were *HbAACT3, HbDXR, HbCMK, HbMDS1, HbMDS2, HbFDPS* and *HbHRT2*. Of these, the expressions of *HbFDPS* and *HbHRT2*, were down-regulated in the latex of CATAS7-33-97 during latex flow while they were up-regulated in the latex of CATAS8-79 at the late stage of latex flow. Moreover, five genes were individually changed only in one clone. For CATAS8-79, the expression of *HbDXR, HbMDS1* and *HbMDS2* were up-regulated at the late stage of latex flow. For CATAS7-33-97, the expression of *HbAACT3* was up-regulated while *HbCMK* was down-regulated at the late stage of latex flow.

### Expression Pattern of Jasmonate and Ethylene Signaling-Related Genes during Latex Flow

**FIGURE 5:**
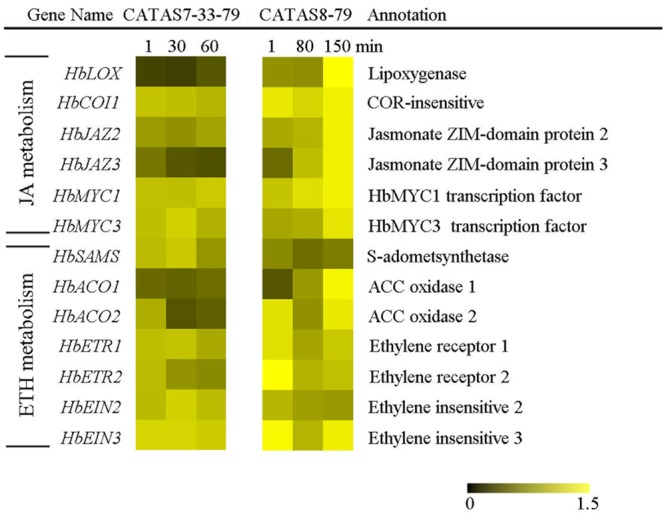
The qRT-PCR analysis of hormone metabolism-related genes across three stages of latex flow between CATAS7-33-97 and CATAS8-79.

It is known that phytohormones play a key role in increasing the natural rubber production (Zhu and Zhang, 2009; Zhao et al., 2011). The differential expressions of six jasmonate signaling-related genes and seven ethylene signaling-related genes during latex flow were revealed between the rubber tree clone CATAS-7-33-97 and CATAS8-79 (**Figure 5** and **Supplementary Figure S1**). In comparison with the rubber tree clone CATAS7-33-97, all five jasmonate signaling-related genes, *HbCOI1, HbJAZ2, HbJAZ3, HbMYC1* and *HbMYC3*, were significantly up-regulated in the latex of the rubber tree clone CATAS8-79 at the late stage of latex flow. The expression pattern of *HbCOI1* and *HbJAZ3* was reversed between the two clones during latex flow. They were down-regulated in the latex of CATAS7-33-97, while they were up-regulated in the latex of CATAS8-79 at the late stage of latex flow. Furthermore, *HbJAZ2, HbMYC1* and *HbMYC3* showed no change in the latex of CATAS7-33-97 while they were up-regulated in the latex of CATAS8-79 at the late stage of latex flow. Among the seven ethylene signaling-related genes, *HbACO1, HbACO2, HbETR2* and *HbEIN3* showed no change in the latex of CATAS7-33-97 while they were up-regulated in the latex of CATAS8-79 at the late stage of latex flow. Similarly, *HbSAMA* was down-regulated during latex flow in the latex of CATAS7-33-97 while they were up-regulated in the latex of CATAS8-79 at the late stage of latex flow.

### Expression Patterns of ROS Generation and Scavenging System Related Genes during Latex Flow

**FIGURE 6:**
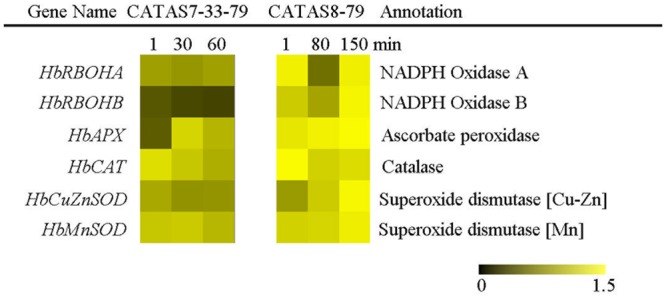
The qRT-PCR analysis of ROS generation and scavenging system related genes across three stages of latex flow between CATAS7-33-97 and CATAS8-79.

The balance of ROS generation and scavenging in laticifer cells influenced the state of latex exploitation (Zhang et al., 2017). The expression of two ROS generation-related genes (*HbRBOHA* and *HbRBOHB*) showed no change in the latex of CATAS7-33-97, while they were up-regulated in the latex of CATAS8-79 at the late stage of latex flow (**Figure 6** and **Supplementary Figure S1**). The expression level of *HbRBOHA* at the early stage of latex flow in the latex of CATAS8-79 was significantly higher than that in the latex of CATAS7-33-97. Among the four ROS scavenging-related genes (*HbAPX, HbCAT, HbCuZnSOD* and *HbMnSOD*), *HbAPX* and *HbCAT* were down-regulated in the latex of CATAS7-33-97, while they showed no change (*HbAPX*) or were up-regulated (*HbCAT*) in the latex of CATAS8-79 at the late stage of latex flow. In contrast to *HbMnSOD* and *HbCuZnSOD* which were not reprogrammed by latex flow in CATAS7-33-97, the two genes were activated the latex of CATAS8-79 at the late stage of latex flow.

### Expression Pattern of Latex Coagulation- Related Genes during Latex Flow

**FIGURE 7:**
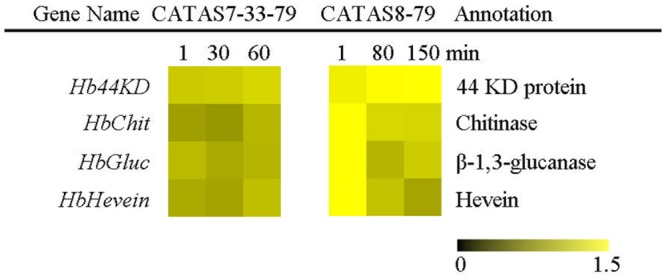
The qRT-PCR analysis of latex coagulation related genes across three stages of latex flow between CATAS7-33-97 and CATAS8-79.

The expression pattern of four latex coagulation-related genes, *Hb44KD, HbChit, HbGluc* and *HbHevein*, were different between rubber tree clones CATAS7-33-97 and CATAS8-79 (**Figure 7** and **Supplementary Figure S1**). There were slight changes in the expression levels of the four genes among the three stages of latex flow in the latex of CATAS7-33-97. By contrast, the *Hb44KD* gene was up-regulated, while all three genes, including *HbChit, HbGluc* and *HbHevein*, were down-regulated during latex flow in the latex of CATAS8-79.

## Discussion

**FIGURE 8:**
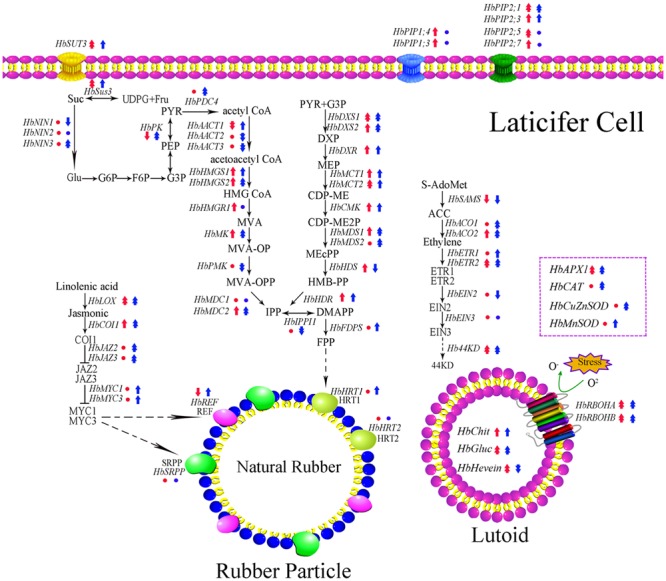
Schematic representation of expression pattern of latex metabolism-related genes in laticifer cell. The genes expression pattern in early or late stage of latex flow between two clones is obtained by CATAS7-33-97 versus CATAS8-79. Red represented early stage of latex flow while blue represented late stage of latex flow. Circle, single arrow, double arrow represented no change, 0.05, 0.01 significant difference. Up-regulation showed high expression in CATAS8-79 and down-regulation showed high expression in CATAS7-33-97.

Transcription and translation are two crucial steps that determine the transfer of genetic information from genes to protein. It is known that the transcript level of a gene can largely reflect its translation state based on the central dogma. In the rubber tree, studies show the expression level of *HbAPX, HbSUT3* and *HbNIN2* is positively related to its protein activity both *in vivo* and *in vitro* (Tang et al., 2010; Liu et al., 2015; Chao et al., 2015b). The duration of latex flow and latex regeneration are two factors that determine the rubber yield of rubber trees (Serres et al., 1994; Chao et al., 2015a). After tapping, latex exploitation usually lasts 10 min to a few hours. However, most of the investigation on the latex metabolism focuses on latex samples after latex flow and neglect changes during latex flow (Tang et al., 2010; He, 2013; Xiao et al., 2014; An et al., 2015; Chao et al., 2015b; Liu et al., 2015; Long et al., 2015; Putranto et al., 2015; Deng et al., 2016; Shi et al., 2016; Makita et al., 2017). Considering that the expression of some candidate reference genes in the latex of the rubber tree are influenced by latex flow (Chao et al., 2016), the expression of latex metabolism-related genes should be influenced in this process. The rubber tree clones CATAS7-33-97 and CATAS8-79 have a different latex flow duration and latex regeneration (Chao et al., 2016). Accordingly, 62 latex metabolism-related genes are differentially expressed during latex flow between the two clones (**Figure 8**). Of these, the expression levels of 38 genes were higher in the latex of CATAS8-79 than that in the latex of CATAS7-33-97 at the early stage of the latex flow (1 min). At the late stage of the latex flow, there were 45 genes with higher expression levels in the latex of CATAS8-79 (**Supplementary Figure S1**). The changes in the gene expression at the late stage should be caused by the current latex flow. The expression status at the early stage should mainly be the state of latex regeneration after latex flow caused by the last tapping, though the slight influence caused by the morphological difference between two clones could not be excluded.

The latex regeneration ability is a crucial factor that determines latex production. In our previous research, the duration of latex flow and latex production are notably different between CATAS8-79 and CATAS7-33-97 (Chao et al., 2016). Here, we further show the differential changes in the rubber content during latex flow between the two clones (**Figure 1**). The high rubber content remains unchangeable within 80 min after tapping in CATAS8-79, but significantly decreases at 30 min after tapping in CATAS7-33-97, suggesting that the rubber tree clone CATAS8-79 is more effective in latex regeneration than CATAS7-33-97. Carbohydrate metabolism, such as sucrose metabolism and glycolysis, not only supplies energy but also generates a carbon skeleton for the formation of organic compounds, including natural rubber (Tang et al., 2010). The SUT and NIN or sucrose synthase take part in sucrose transport and decomposition or synthesis, respectively (Zhang C. et al., 2016). Recently, Liu et al. (2015) cloned three invertases (*HbNIN1-3*) in the latex of the rubber tree and identified that *HbNIN2* is the crucial isoform for latex regeneration. In the current research, only *HbNIN3* was up-regulated at the late stage of latex flow in the latex of CATAS8-79 compared with that of CATAS7-33-97 (**Figure 8**). Moreover, the expression levels of *HbSUT3* and *HbSus3* at both the early and late stage of latex flow in the latex of CATAS8-79 are significantly higher than that of CATAS7-33-97 (**Figure 8**). The results suggest that sucrose synthesis and transport rather than decomposition might be the rate-limiting steps for latex regeneration, and *HbNIN3* should play a key role in initiating the regeneration of latex after tapping. PK and pyruvate dehydrogenase complex (PDC) are major contributors to the control of glycolysis (Fleige et al., 2007; Megguer et al., 2017). In contrast to CATAS7-33-97, both *HbPK* and *HbPCD4* are significantly activated at the late stage of latex flow in the latex of CATAS8-79 (**Figure 8**). This might provide much of the acetyl coenzyme-A for synthesis of IPP, the direct precursor of natural rubber, in the rubber tree clone CATAS8-79. The cytoplastic MVA pathway is the main enzymatic reaction for IPP biosynthesis (Kang et al., 2016). In the MVA pathway, HMG CoA is synthesized though 3-hydroxy-3-methylglutaryl coenzyme-A synthase (HMGS) and then reduced to MVA by 3-hydroxy-3-methylglutaryl coenzyme-A reductase (HMGR). It has long been known that HMGR is the key rate-limiting enzyme of the MVA pathway in many species, such as *Homo*, yeast, and Arabidopsis (Omkumar et al., 1994; Dale et al., 1995; Burg et al., 2008). In the rubber tree, four *HbHMGR*s have been cloned, and *HbHMGR1* was previously recognized as the key member involved in natural rubber biosynthesis (Sando et al., 2008). In the present study, its expression pattern is similar between CATAS7-33-97 and CATAS8-79. In contrast, the expression levels of *HbHMGS1* and *HbHMGS2* at the early and late stage of latex flow in the latex of CATAS8-79 are significantly higher than those in the latex of CATAS7-33-97 (**Figure 8**). It seems that the biosynthesis of HMG CoA by HMGS is more important than the biosynthesis of MVA by HMGR for enhanced natural rubber biosynthesis in the rubber tree. In addition to the MVA pathway, the plastidic MEP pathway is supposed to act as an alternative pathway for IPP biosynthesis in the rubber tree (Chow et al., 2012). In the present study, most of the tested genes involved in the MEP pathway are activated while only three of the ten genes in the MVA pathway (*HbHMGS1-2* and *HbMK*) are activated at both the early and late stages of latex flow in the latex of CATAS8-79 (**Figure 8**). The overall activation of the MEP pathway in CATAS8-79 may supply much more IPP for natural rubber biosynthesis. Jasmonates have important roles in the regulation of secondary metabolite biosynthesis (Zhou and Memelink, 2016). In the present research, it is of interest that in comparison with the expression pattern of CATAS7-33-97, all jasmonate biosynthesis and signaling-related genes are activated at the late stage of latex flow in the latex of CATAS8-79. This activation may contribute to enhanced rubber biosynthesis between two tappings in CATAS8-79.

The duration of latex flow is a factor that determines latex production. The duration of the latex flow of CATAS8-79 is notably longer than that of CATAS7-33-97 (Chao et al., 2016). The phloem turgor pressure is recognized as the initial power of latex exploitation after tapping. In laticifer cells, water as well as natural rubber accumulation causes a huge turgor pressure (∼10 Pa) (An et al., 2014). Aquaporins are a group of proteins that mediate the trans-membrane transport of water and other small solutes. The PIP is one subfamily of aquaporins and plays a crucial role in laticifers water transport in the rubber tree (Zou et al., 2015). The transcript levels of all the genes at the early stage of latex flow in CATAS8-79 are higher than that in CATAS7-33-97, suggesting that water entrance into laticifer cells is more active in CATAS8-79 than in CATAS7-33-97. The available data show that *HbPIP2;1* is important for water metabolism (Tungngoen et al., 2009). In the present study, *HbPIP1;3* and *HbPIP1;4* exhibit high transcript abundance at the early stage of latex flow but low expression abundance at the late stage of latex flow in CATAS8-79 (**Figure 2**). By contrast, the transcript level of *HbPIP2;1* is very low during latex regeneration (at the early stage of latex flow) in both CATAS7-33-97 and CATAS8-79, while they are up-regulated especially in CATAS8-79 at the late stage of latex flow. This differential expression pattern suggests that HbPIP1;3 and HbPIP1;4 play a crucial role in the process of latex regeneration while HbPIP2;1 may be active in regulating water entrance into laticifer cells during latex flow. In natural rubber production, Ethrel (an ethylene releaser) is widely used to increase rubber yield per tapping by prolonging the duration of latex flow (Zhu and Zhang, 2009). Additionally, ACO is a key enzyme that catalyzes the last step of ethylene biosynthesis, while the ETR is essential for ethylene signaling (Hall and Bleecker, 2003; Sun et al., 2017). Here, we show that the expression levels of *HbACO2* and *HbETR2* at both early and late stages of latex flow in the latex of CATAS8-79 are significantly higher than that of CATAS7-33-97, hinting that ethylene production and ethylene signaling are more active in the latex of CATAS8-79. The activation of ethylene signaling in CATAS8-79 may contribute to the longer duration of latex flow. It may also be related to the up-regulation of *HbRBOHA* and *HbRBOHB* at both the early and late stages of latex flow in the latex of CATAS8-79. The available data show that ethylene signaling has a synergistic role with ROS generation (Zhang H. et al., 2016; Zhang M. et al., 2016). In Arabidopsis, ERF74, an ERF, can bind to the promoter of RbohD and activate its expression (Yao et al., 2017). In rice, OsEIL1, a rice homologue of AtEIN3 and can bind to the promoters of OsRbohA and OsRbohB directly (Yang et al., 2017). The RBOH located at the surface of the lutoids is the main source of ROS, while antioxidant proteins (CAT, SOD, APX) play a key role in scavenging ROS in the latex (Zhang et al., 2017). In the current research, it is clearly shown that compared with other ROS scavenging genes (*HbAPX, HbCuZnSOD* and *HbMnSOD*), *HbCAT* exhibits an opposite expression pattern during latex flow in both clones (**Figure 6**). Compared with the high expression level of *HbCAT* at the early stage of latex flow, the transcript abundance of the gene is low at the late stage in both rubber tree clones. We thus deduce that the expression level of *HbCAT* should be up-regulated until the next tapping and the gene may play a key role in latex regeneration. Since CAT has a lower affinity for H_2_O_2_ than APX, the enzyme is effective only in the presence of a massive level of H_2_O_2_ (Anjum et al., 2016). The higher abundance of *HbCAT* in CATAS8-79 suggests that the clone has a high concentration of H_2_O_2_ in laticifer cells, which may be the main reason for CATAS8-79 having a higher rate of tapping panel dryness during production (Li and Liu, 2014). The activation of *HbAPX, HbCuZnSOD* and *HbMnSOD* at the late stage of latex flow in the latex of CATAS8-79 may contribute to scavenging ROS and maintaining latex exploitation. On the other hand, the plug formation at the end of the severed laticifers results in the termination of the latex flow. Since the fractured lutoid effectively causes latex coagulation, it is traditionally believed that the ethylene-caused prolongation of the latex flow duration is associated with the increased stability of lutoids (Wititsuwannakul et al., 2008). Recently, we demonstrate that the ethylene-caused prolongation of latex flow is associated with increased levels of a 44 kDa protein in the C-serum (Shi et al., 2016). The protein acts as a universal antagonist of rubber particle aggregation that is caused by proteins from the lutoids. These proteins include hevein, chitinase and glucanase. The hevein, glucanase and the combination of chitinase and glucanase are effective in aggregating rubber particles (Gidrol et al., 1994; Wititsuwannakul et al., 2008; Wang et al., 2013), which contribute to plug formation at the end of the severed laticifers. In the present study, the expression level of *Hb44KD* at any stage of latex flow in the CATAS8-79 latex is higher than that in CATAS7-33-97 latex. By contrast, the expression levels of the latex coagulation-related genes, *HbChit, HbCluc* and *HbHevein*, are up-regulated at the late stage of latex flow in CATAS7-33-97 latex, while there was no change or down-regulation in CATAS8-79.

Taken together, the expression levels of 62 latex metabolism related genes were monitored in two rubber tree clones with a differential latex regeneration and latex flow duration. It is speculated that the up-regulation of the antagonist *Hb44KD*, aquaporin *HbPIP2;1*, ethylene biosynthesis key gene *HbACO1*, and the down-regulation of the coagulation factors *HbChit, HbGluc* and *HbHevein*, are the key members that determine the duration of latex flow after tapping. While the up-regulation of the aquaporins *HbPIP1;3* and *HbPIP1;4*, the sucrose metabolism members *HbSUT3* and *HbSus3*, the key MVA pathway members *HbHMGS1-2* and *HbMK*, and the activation of the MEP pathway and jasmonate pathway, are suggested to play key roles in promoting latex regeneration between two tappings. A schematic representing the molecular difference of latex metabolism between CATAS7-33-97 and CATAS8-79 is displayed (**Figure 8**), which provides some clues for revealing the regulation of latex metabolism and the benefits of molecular breeding in the rubber tree.

## Author Contributions

JC designed and carried out the experiment of this study, and wrote the manuscript. SY and YC participated and analyzed data in the experiment. W-MT planned the study and participated in the design of the experiment. All authors have read and approved the manuscript in its final form.

## Conflict of Interest Statement

The authors declare that the research was conducted in the absence of any commercial or financial relationships that could be construed as a potential conflict of interest.

## Supplementary Material

FIGURE S1 The qRT-PCR analysis of all latex metabolism related genes across three stages of latex flow between CATAS7-33-97 and CATAS8-79. The capital letter represents *p* < 0.01 while lower case represents *p* < 0.05. The same letter indicated no significant difference among groups.

## Abbreviations

AACT: Acetyl coenzyme A acetyltransferase
ACO: 1-aminocyclopropane-1-carboxylate oxidase
APX: L-ascorbate peroxidase
CAT: catalase
Chit: chitinase
CMK: 4-(cytidine 5-diphospho)-2-*C*-methyl-D-erythritol kinase
COI: coronatine insensitive
DXR: 1-deoxy-D-xylulose 5-phosphate reductoisomerase
DXS: 1-deoxy-D-xylulose 5-phosphate synthase
ERF: ethylene response factor
ETH: ethylene
ETR: ethylene receptor
EIN: ethylene insensitive
FDPS: farnesyl diphosphate synthase
Gluc: glucanase
HDR: 4-hydroxy-3-methylbut-2-enyl diphosphate reductase
HDS: 4-hydroxy-3-methylbut-2-enyl diphosphate synthase
HMGR: 3-hydroxy-3-methylglutaryl-coenzyme A reductase
HMGS: 3-hydroxy-3-methylglutaryl-coenzyme A synthase
HRT: Hevea rubber transferase
IPP: isopentenyl pyrophosphate
IPPI: isopentenyl pyrophosphate isomerase
JA: jasmonic acid
JAZ: jasmonate-ZIM-domain
LOX: lipoxygenase
MCT: 2-*C*-methyl-D-erythritol 4-phosphate cytidylyltransferase
MDC: Diphosphomevalonate decarboxylase
MDS: 2-*C*-methyl-D-erythritol 2,4-cyclodiphosphate synthase
MEP: 2-*C*-methyl-D-erythritol 4-phosphate
MVA: mevalonate
MK: mevalonate kinase
NIN: alkaline/neutral invertase
PDC: pyruvate decarboxylase
PIP: plasma membrane intrinsic protein
PK: pyruvate kinase
PMK: Phosphomevalonate kinase
qRT-PCR: quantitative real-time PCR
RBOH: respiratory burst oxidase homolog
REF: Rubber elongation factor
ROS: reactive oxygen species
SAMS: *S*-adenosylmethionine synthetase
SOD: superoxide dismutase
SRPP: small rubber particle protein
Sus: sucrose synthase
SUT: sucrose transporter
UBC2b: ubiquitin-protein ligase (AtUBC2)
