# Supplementary figures and images for: Transcript Profiling of Hevea brasiliensis during Latex Flow
Source: Front Plant Sci. 2017 Nov 7;8:1904. doi: 10.3389/fpls.2017.01904 (PMC5682034; doi:10.3389/fpls.2017.01904)

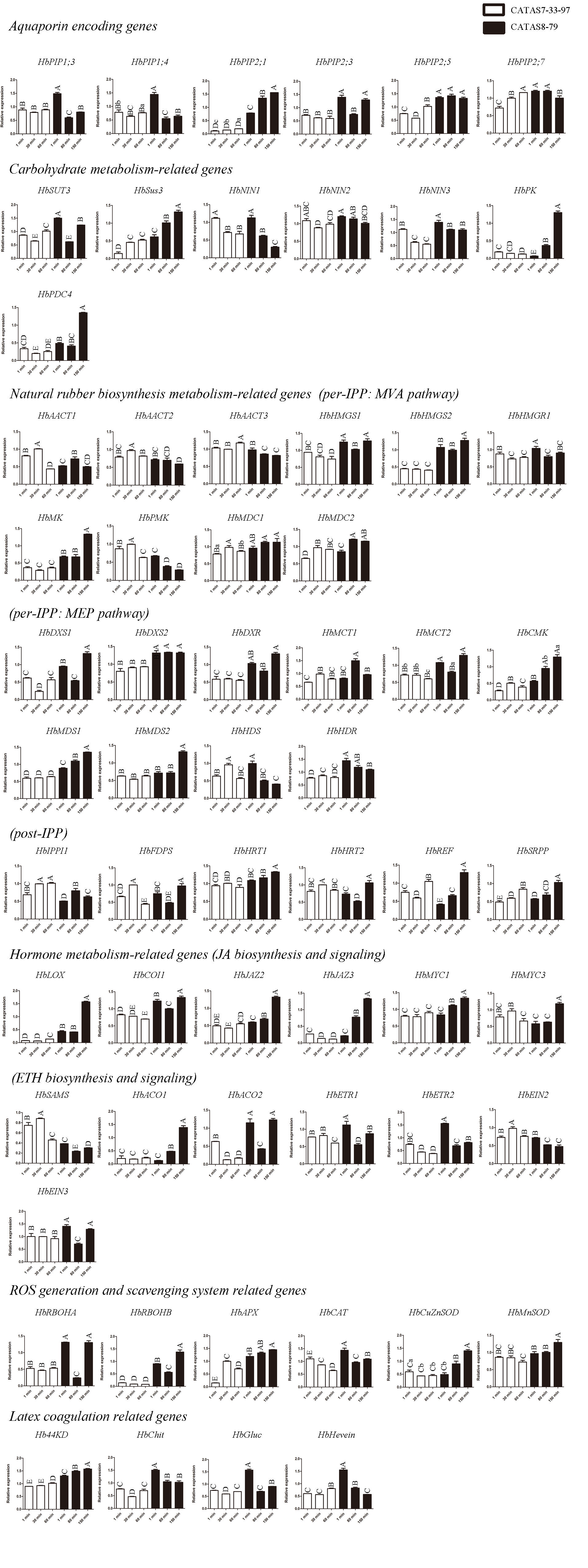

Supplement: FIGURE S1 — The qRT-PCR analysis of all latex metabolism related genes across three stages of latex flow between CATAS7-33-97 and CATAS8-79. The capital letter represents p < 0.01 while lower case represents p < 0.05. The same letter indicated no significant difference among groups. [file Image_1.TIF]
