# Supplementary material for: Transcript Profiling of Hevea brasiliensis during Latex Flow
Source: Front Plant Sci. 2017 Nov 7;8:1904. doi: 10.3389/fpls.2017.01904 (PMC5682034; doi:10.3389/fpls.2017.01904)
Supplement: Supplementary file 2 [file Table_1.DOCX]

**Additional file 1**

**Table S1. Primers used in this paper.**

| Gene Name | | Primer sequence | Annotation | Amplification  Efficiency | | GenBank Accession | | Reference | |
| --- | --- | --- | --- | --- | --- | --- | --- | --- | --- |
| Water transport-related genes | | | | | | | | | |
| *HbPIP1;3* | Forward: AGCCCAGACTGATAAGGACTACAA  Reverse: GATGAACTCGGCAATCCCAG | | Plasma membrane intrinsic protein 1;3 | | 83.5% | | XM_021807425.1 | Zou et al. 2015 | |
| *HbPIP1;4* | Forward: CCACCTGCTCCCTTGTTTGA  Reverse: GATGAACTCGGCAATCCCAG | | Plasma membrane intrinsic protein 1;4 | | 100.6% | | GQ479823.1 | Zou et al. 2015 | |
| *HbPIP2;1* | Forward: TTACAAGAGCCAAACCGACCC  Reverse: GCGATACCAAGAATCCCAACAC | | Plasma membrane intrinsic protein 2;1 | | 86.3% | | FJ851079.1 | Tungngoen et al. 2009 | |
| *HbPIP2;3* | Forward: GCCGAGTTCATTGCTACCCT  Reverse: CAAGAATACCAACACCACCACA | | Plasma membrane intrinsic protein 2;3 | | 81.8% | | XM_021806524.1 | Zou et al. 2015 | |
| *HbPIP2;5* | Forward: CATCAACCCAGCCAGGAGTCTA  Reverse: CACCAATGAAGGGACCAACC | | Plasma membrane intrinsic protein 2;5 | | 100.7% | | KF921089.1 | Zou et al. 2015 | |
| *HbPIP2;7* | Forward: CATATTAACCCGGCGGTCAG  Reverse: CACCAACCCAACACCACAAA | | Plasma membrane intrinsic protein 2;7 | | 85.5% | | GQ479824.1 | Zou et al. 2015 | |
| Carbohydrate metabolism-related genes | | | | | | | | | |
| *HbSUT3* | Forward: CACCACAACCACCATCAC  Reverse: GTGGAAGAGGTTCAGAAGAG | | Sucrose transporter 3 | | 99.3% | | EF067334.2 | | Tang et al., 2010 |
| *HbSus3* | Forward: GCTGAATCTGTTCCTCT  Reverse: TCACAACTCCGAAACTT | | Sucrose synthase 3 | | 96.9% | | KC492045.1 | | Xiao et al., 2014 |
| *HbNIN1* | Forward: AAGAGGCAATCATGCAAACAA  Reverse: TGATAAAGGGCAAAGAATGGTG | | Alkaline/Neutral invertase 1 | | 99.5% | | GU573728.1 | | Liu et al., 2015 |
| *HbNIN2* | Forward: GAAGAGAGGCAAACAAACAAG  Reverse: CGAAACCAAACATTTCTCACTTTAC | | Alkaline/Neutral invertase 2 | | 88.7% | | GU573727.1 | | Liu et al., 2015 |
| *HbNIN3* | Forward: ATGGTTTATTATTGTGGCAGTCCGA  Reverse: TCCGAACAATATCATACTCTCCCTT | | Alkaline/Neutral invertase 3 | | 93.4% | | KC577600.1 | | Liu et al., 2015 |
| *HbPK* | Forward: ACTCACATCAGCCACTG  Reverse: ACTTTCCCTAAACCCTC | | Pyruvate kinase | | 85.2% | | MF489241 | | In this study |
| *HbPDC4* | Forward: CCAAGGTCCAATGTGAGGAG  Reverse: ATCTTGTCCTGAAAGAACAAAGGA | | Pyruvate decarboxylase 4 | | 89.5% | | KJ599635.1 | | Long et al., 2015 |
| Natural rubber biosynthesis-related genes | | | | | | | | | |
| *HbAACT1* | Forward: GCATTGGCTATACCTAAAACGATTG  Reverse: GCGAAGGCTTCATTTATTTCATAGT | | Acetyl coenzyme A acetyltransferase 1 | | 87.5% | | JN036529.1 | | Deng et al., 2016 |
| *HbAACT2* | Forward: GCTCCGACCAAGTTTCAAAGAG  Reverse: ATTACAGCAGCAGCACCATCAC | | Acetyl coenzyme A acetyltransferase 2 | | 83.0% | | AB294687.1 | | Deng et al., 2016 |
| *HbAACT3* | Forward: GCATCTGCCCTTGTTCTTGAG  Reverse: GCTGATGTTAGAGTGACCAATTTGA | | Acetyl coenzyme A acetyltransferase 3 | | 100.9% | | AF429383.1 | | Deng et al., 2016 |
| *HbHMGS1* | Forward: CGGGTGACACTGTTCTCTTATGG  Reverse: GGGATGTTGGCCTTCATGTAGT | | 3-Hydroxy-3-methylglutaryl-coenzyme A synthase 1 | | 85.7% | | JN036533.1 | | Deng et al., 2016 |
| *HbHMGS2* | Forward: CTTGGCGCCTGGAACATACT  Reverse: TGTATCGCCAACAGCCTTCTG | | 3-Hydroxy-3-methylglutaryl-coenzyme A synthase 2 | | 91.1% | | AY534617.1 | | Deng et al., 2016 |
| *HbHMGR1* | Forward: GCTGTTATATGAAGTATGGAGATAGG  Reverse: AAGGGTAGAGAGAGAAGTAGAGG | | 3-Hydroxy-3-methylglutaryl-coenzyme A reductase 1 | | 83.4% | | AB294692 | | Deng et al., 2016 |
| *HbMK* | Forward: GTCTGGTAATCTGACTCGCATCAA  Reverse: GTTCCTCCCAACTCTTGTGTTAGTG | | Mevalonate kinase | | 99.9% | | JN036543.1 | | Deng et al., 2016 |
| *HbPMK* | Forward: GAAATGCCATGCTTCAGATCAG  Reverse: TGATTCAGGCTCTATCGGAACA | | Phosphomevalonate kinase | | 88.9% | | JN036535.1 | | Deng et al., 2016 |
| *HbMDC1* | Forward: CACACTTAACCTGTGCTGATAGTAATC  Reverse: TGAGGTGTTCCTACAGAACGATTC | | Diphosphomevalonate decarboxylase 1 | | 100.8% | | JN036538.1 | | Deng et al., 2016 |
| *HbMDC2* | Forward: GTAATAGGCAGGCTGCTGCCCG  Reverse: CTGGACCTCGACCTGGTCTTGTG | | Diphosphomevalonate decarboxylase 2 | | 87.5% | | JN036539.1 | | Deng et al., 2016 |
| *HbDXS1* | Forward: CAGAAGCAGAAGTGGACAAGGAT  Reverse: AACGGCGAAGGAAGAGATTTAAG | | 1-deoxy-D-xylulose 5-phosphate synthase 1 | | 96.6% | | AY502939.1 | | Deng et al., 2016 |
| *HbDXS2* | Forward:TGGTTTGGTTGGTGCAGATG  Reverse: CTACCATGTTGGGCAAGCAA | | 1-deoxy-D-xylulose 5-phosphate synthase 2 | | 81.1% | | DQ473433.1 | | Deng et al., 2016 |
| *HbDXR* | Forward: ACCCTTCTATGGATCTTGCCTATG  Reverse: GCAGCACTAAGCACTCCAGTCA | | 1-deoxy-D-xylulose 5-phosphate reductoisomerase | | 85.0% | | DQ437514.1 | | Deng et al., 2016 |
| *HbMCT1* | Forward: CAGCTGCGAAAGAGAAGAGTGTT  Reverse: GCTTGCACCCATTCTTTTGC | | 2-C-methyl-D-erythritol 4-phosphate cytidylyltransferase 1 | | 87.7% | | AB294702.1 | | Deng et al., 2016 |
| *HbMCT2* | Forward: CCAAAGAACGGGAGGAAGAGA  Reverse: AGGTTCATGCGAAGAAGATGATG | | 2-C-methyl-D-erythritol 4-phosphate cytidylyltransferase 2 | | 91.5% | | AB294703.1 | | Deng et al., 2016 |
| *HbCMK* | Forward: GGCAAGACCGCCACTGAA  Reverse: CAGGGCTTGGTGGTGGAA | | 4-(cytidine 5-diphospho)-2-C-methyl-D-erythritol kinase | | 100.6% | | XM_021808894.1 | | Deng et al., 2016 |
| *HbMDS1* | Forward: AACTTGGATGCCACCTTGATTC  Reverse: AGCTGACACAAATTGTCCCTGAT | | 2-C-methyl-D-erythritol 2,4-cyclodiphosphate synthase 1 | | 94.6% | | FJ196164.1 | | Deng et al., 2016 |
| *HbMDS2* | Forward: GGATGCCACCTTAATTCTTCAAAG  Reverse: CCAAGCAGCTGACACAAATTGT | | 2-C-methyl-D-erythritol 2,4-cyclodiphosphate synthase 2 | | 83.4% | | AY502938.1 | | Deng et al., 2016 |
| *HbHDS* | Forward: GCTGACAAAGCCATTACCCAAT  Reverse: CGTGTACCTTCTGGCAAAAGC | | 4-hydroxy-3-methylbut-2-enyl diphosphate synthase | | 89.6% | | AB294707.1 | | Deng et al., 2016 |
| *HbHDR* | Forward: TTCCTACCAGAAGGTCCCATTAC  Reverse: ATATCCAACCACTAATTGCAGCTC | | 4-hydroxy-3-methylbut-2-enyl diphosphate reductase | | 97.9% | | AB294708.1 | | Deng et al., 2016 |
| *HbIPPI* | Forward: ACCTTGGTTTAGACTAGTTGTGGAC  Reverse: AACTCGTTTACAACTGACATTACCA | | Isopentenyl diphosphate isomerase 1 | | 90.2% | | AB294696.1 | | Deng et al., 2016 |
| *HbFDPS* | Forward: AATGGGTTGAGCGGATGTTGGA  Reverse: TCCTGGGAATGTGATTGCGAAG | | Farnesyl diphosphate synthase | | 87.4% | | AY349419.1 | | Deng et al., 2016 |
| *HbHRT1* | Forward: GCAAATGCAACTGGAAGCGG  Reverse: AGACGGGTCTCCCCAGAAGT | | Hevea rubber transferase 1 | | 92.2% | | LOC110667933 | | Chao et al., 2015 |
| *HbHRT2* | Forward: TTTCTGTTCCTAGCTCATCCTGCCT  Reverse: TGCATGTCGGAATTTGCTACAATAC | | Hevea rubber transferase 2 | | 90.7% | | LOC110667932 | | Chao et al., 2015 |
| *HbREF* | Forward: ACCAAGAGACTTTCTAAGGTGCTA  Reverse: CACTTCATCATCTTACTCAACTGG | | Rubber elongation factor | | 89.7% | | AY430052.2 | | Deng et al., 2016 |
| *HbSRPP* | Forward: TATTCCACATCCAAAACACACCACC  Reverse: TGGAAATGGTACAACAGAGTTGC | | Small rubber particle protein | | 89.2% | | HQ640231 | | Deng et al., 2016 |
| Jasmonate and ethylene signaling-related genes | | | | | | | | | |
| *HbLOX* | | Forward: TCTCCATGCAGCAATCA  Reverse: GGGAAATGCCAACAACA | Lipoxygenase | | 92.5% | MF489242 | | In this study | |
| *HbCOI1* | | Forward: GGGTTCCTTCAATGATATGCCAG  Reverse: TGGTCCTCTGTGTCAAGCAATG | Coronatine insensitive | | 100.4% | EU136026.1 | | He, 2013 | |
| *HbJAZ2* | | Forward: AGTGGCTGCTTAATCTTCGCA  Reverse: ACCAAAAGGAAACAGCAGGATT | Jasmonate-ZIM-domain 2 | | 98.0% | KJ001643.1 | | He, 2013 | |
| *HbJAZ3* | | Forward: AACTTGCCCAAAATGGAATCTC  Reverse: ACATACGGGGATACCGAAGTCA | Jasmonate-ZIM-domain 3 | | 87.0% | KP844628.1 | | He, 2013 | |
| *HbMYC1* | | Forward: TCTTTAAATAACCCCAAGTCTCACTT  Reverse: TGTTCTTTTCTGTTTTTTGGTTTAGC | MYC1 | | 88.9% | GU434304.1 | | He, 2013 | |
| *HbMYC3* | | Forward: TCTCTCGCTTCCCTACCCTCT  Reverse: TCCTCCATTGATATCTTTTTCTCG | MYC3 | | 85.9% | HM347338.1 | | He, 2013 | |
| *HbSAMS* | | Forward: CACCAATGGCATAGGAGAC  Reverse: ACCTTAACCCGTCAGGGC | S-Adenosylmethionine synthetase | | 87.4% | XM_021820469.1 | | Putranto et al., 2015 | |
| *HbACO1* | | Forward: TGCAGGCCTCAAGTTCCAA  Reverse: CTTCATCATCAATAGCGGCT | 1-aminocyclopropane-1-carboxylate oxidase 1 | | 92.3% | CAN85569.1 | | Putranto et al., 2015 | |
| *HbACO2* | | Forward: CAGCACCAGCTTTGG  Reverse: ACACTCCCAAATATTCCTCTC | 1-aminocyclopropane-1-carboxylate oxidase 2 | | 94.1% | XM_021794640.1 | | Putranto et al., 2015 | |
| *HbETR1* | | Forward: GGTAATGCTGTCAAGTTCAC  Reverse: GGGCTGAGTGAATTTAGT | Ethylene Receptor 1 | | 100.6% | XM_021800366.1 | | Putranto et al., 2015 | |
| *HbETR2* | | Forward: TGCCCTGACATCAAGTGCT  Reverse: TCAGACCCCAAAACCGAAG | Ethylene Receptor 2 | | 100.2% | XM_021794055.1 | | Putranto et al., 2015 | |
| *HbEIN2* | | Forward: TTGCCATATCTTGCCGAAAG  Reverse: GCCCTATATTTACCCAGTTGAG | Ethylene insensitive 2 | | 85% | XM_021804758.1 | | Putranto et al., 2015 | |
| *HbEIN3* | | Forward: CAATGTCCTTACAGCCAAC  Reverse: AGTTCACCATCGGGACAG | Ethylene insensitive 3 | | 85.6% | XM_021804758.1 | | Putranto et al., 2015 | |
| ROS generation and scavenging system related genes | | | | | | | | | |
| *HbRBOHA* | | Forward: CCATTTTCCATCACTTCTGC  Reverse: TTGTTTCCTTGTAAGCCCTC | Respiratory burst oxidase homolog A | | 87.4% | XM_021784436.1 | | Putranto et al., 2015 | |
| *HbRBOHB* | | Forward: TGTGCTGTTACTAGTTGGTC  Reverse: CAGAGTCTTCTTTCGTTTTG | Respiratory burst oxidase homolog B | | 95.9% | XM_021825486.1 | | Putranto et al., 2015 | |
| *HbAPX* | | Forward: ATTGCTGATCCCGTCTT  Reverse: GTCGGCACCATCCTCTA | L-ascorbate peroxidase | | 94.1% | XM_021783808.1 | | Chao et al., 2015b | |
| *HbCAT* | | Forward: TATAGATCCTGGGCACCTG  Reverse: GGTGGCATCATCTTCAAATG | Catalase | | 92.2% | XM_021810359.1 | | Putranto et al., 2015 | |
| *HbCuZnSOD* | | Forward: AGACACAACAAATGGCTGC  Reverse: TGAGTGAAGGTCTTGTAAC | Superoxide dismutase [Cu-Zn] | | 80.7% | AF457209.1 | | Putranto et al., 2015 | |
| *HbMnSOD* | | Forward: CTTGGACAAAGAATTGAAGAAGC  Reverse: ATACACTTCACTTGCATACTTCC | Superoxide dismutase [Mn] | | 88.6% | AJ289158.1 | | Putranto et al., 2015 | |
| Latex coagulation-related genes | | | | | | | | | |
| *Hb44KD* | | Forward: GCTAGCTCAGATATGGTTGACTTCC  Reverse: TGATGATTCTTCACCCGTCATCGTA | Probable linoleate 9S-lipoxygenase 5 | | 83.7% | XP_021653020 | | Shi et al., 2016 | |
| *HbChit* | | Forward: CTTCTTCCCGTCCCTTAG  Reverse: CCTTCTTGCCTTGCTTGC | Chitinase | | 100.7% | XP_021671429 | | Shi et al., 2016 | |
| *HbGluc* | | Forward: CTCCAAAGCCTTACCAATCCT  Reverse: ACATCATCCCTGAAAGCACCT | Glucanase | | 94.2% | XP_021637251.1 | | Shi et al., 2016 | |
| *HbHevein* | | Forward: TCATGGCGGAGCAAGTATGGC  Reverse: CACACTCAAGCACTTTCCACAGG | Hevein | | 86.9% | AJ012583.1 | | Shi et al., 2016 | |
| Reference gene | | | | | | | | | |
| *UBC2b* | | Forward: CGACCAAGTTTTCATTTCGGGTG  Reverse: AGTCTCTTCTTTGCTGGGGTTG | Ubiquitin-protein ligase (ATUBC2) | | 82.3% | HQ323247 | | Chao et al., 2016 | |
